# A Review of Non-Powder-Bed Metal Additive Manufacturing: Techniques and Challenges

**DOI:** 10.3390/ma17194717

**Published:** 2024-09-26

**Authors:** Jie Xu, Yifan Fei, Yuanzhe Zhu, Wei Yu, Donggang Yao, Jack G. Zhou

**Affiliations:** 1 Department of Mechanical Engineering and Mechanics, Drexel University, Philadelphia, PA 19104, USA; jx69@drexel.edu (J.X.); yz679@drexel.edu (Y.Z.); wy74@drexel.edu (W.Y.); 2 School of Mechanical Engineering, Nanjing Institute of Technology, Nanjing 211167, China; feiyifan@njit.edu.cn; 3 School of Materials Science and Engineering, Georgia Institute of Technology, Atlanta, GA 30332, USA; yao@gatech.edu

**Keywords:** metal additive manufacturing, direct energy deposition, material extrusion, sheet lamination, manufacturing methods, product quality

## Abstract

Metal additive manufacturing has significantly evolved since the 1990s, achieving a market valuation of USD 6.36 billion in 2022, with an anticipated compound annual growth rate of 24.2% from 2023 to 2030. While powder-bed-based methods like powder bed fusion and binder jetting dominate the market due to their high accuracy and resolution, they face challenges such as lengthy build times, excessive costs, and safety concerns. Non-powder-bed-based techniques, including direct energy deposition, material extrusion, and sheet lamination, offer advantages such as larger build sizes and lower energy consumption but also encounter issues like residual stress and poor surface finish. The existing reviews of non-powder-bed-based metal additive manufacturing are restricted to one technical branch or one specific material. This survey investigates and analyzes each non-powder-bed-based technique in terms of its manufacturing method, materials, product quality, and summary for easy understanding and comparison. Innovative designs and research status are included.

## 1. Introduction

MAM has experienced steady development since the 1990s and has greatly influenced human society in various ways during the last 10 years. In 2022, MAM reached a total valuation of USD 6.36 billion, increasing with a 24.2% compound annual growth rate between 2023 and 2030 [1]. Compared with metal subtractive manufacturing, MAM has great advantages in saving raw materials, creating complex geometries, consolidating parts, and reducing costs. Based on ISO/ASTM 52900, MAM includes five major categories: PBF, BJ, ME, DED, and SL [2]. Among these technologies, PBF and BJ fabricate 3D objects based on a print bed. Currently, powder-bed-based MAM techniques are the most common in the industry, dominating the market share by a total of 70% in 2020, with rates of 54% for PBF and 16% for BJ. Moreover, the market shares for DED, ME, and SL are 16%, 10%, and 2%, respectively [3,4].

Powder-bed-based MAM methods are well known for their ability to fabricate lightweight [5,6] and complicated geometrical parts [5,7,8]. Moreover, high dimensional accuracy and resolution are other features [9,10,11]. However, the disadvantages of powder-bed-based MAM pose great challenges in industrial applications. A long building time and limited build size are the two key factors that limit the additive fabrication process [5,12,13]. In addition, high expenses, such as apparatus and powder costs, limit the large-scale application and development of the metal additive manufacturing market [14,15,16]. Moreover, health and safety issues when handling metal powders remain an increasing concern, and additional attention and management are required to avoid metal powder exposure and explosion risks, since many metal powders are carcinogenic, flammable, and explosive [15,17,18,19]. Compared with other technologies, post-processes in powder-bed-based metal printing can be very costly and complicated. For instance, the de-powdering process is conducted at an automatic cleaning station, where all of the unmelted powder undergoes high-frequency vibration and is eventually separated from the part. However, the process must be carefully designed and considered for hollow structures, as this makes cleaning even more complicated [20,21].

Recently, scholars have reviewed the individual categories within non-powder-bed-based MAM. Liu et al. [22] reviewed the LDED of lightweight aluminum alloys, presenting key challenges, process parameters, suggested strategies, and future research directions. Zhang et al. [23] reviewed the application of ultrasound in DED, highlighting the mechanisms, system configuration, and performance enhancement of ultrasound-assisted DED. Srivastava et al. [24] investigated the processes, materials, and behavior of metals in WAAM, discussing the overview, printing optimization, materials, applications, challenges, and emerging trends in WAAM. Obiko et al. [25] studied the application of WAAM for remanufacturing, repairing, and refurbishing complex structural components. Yin et al. [26] explored the mechanisms of CSAM, with a detailed description of mechanical properties and strengthening techniques. Sadaf et al. [27] explored the ME technology using metallic materials with highly filled polymers, thoroughly discussing the selection of metal powders, binder systems, printing process control, and post-processing techniques. Similarly, Bankapalli et al. [28] provided an in-depth analysis of extrusion-based MAM, covering the entire process from material preparation to final metallic product manufacturing. 

Based on the above investigation, it is evident that most of the existing reviews are restricted to one technical branch, one material, or even one specific application. Therefore, to better promote the application of MAM and fulfill various requirements in the industry, a detailed review targeting major non-powder-bed-based MAM techniques is urgently needed. This review explores non-powder-bed-based MAM technologies, including printing mechanisms, materials, and qualities. In addition, the state of the art of these technologies is investigated. Innovative designs, mechanisms, and system optimizations are presented. By reviewing this survey, readers will obtain a broad view of several advanced MAM technologies, enabling them to select the specific 3D metal printing techniques that best suit their application, thereby enhancing the industrial applicability of MAM. 

## 2. Classification of Non-Powder-Bed-Based MAM

**Figure 1:**
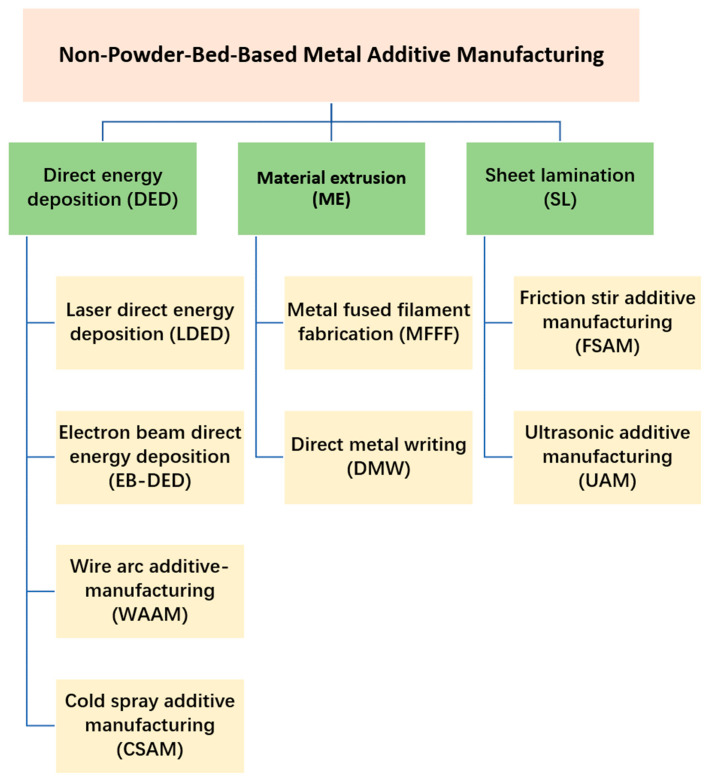
Categories for current major non-powder-bed-based MAM techniques.

The classification of the current non-powder-bed-based MAM techniques discussed in this survey is shown in Figure 1. 

According to ISO/ASTM 52900 [2], DED is an ‘additive manufacturing process in which focused thermal energy is used to fuse materials by melting as they are being deposited’. Based on the mechanism, DED processes can be categorized into LDED [29], EB-DED [30], WAAM, and CSAM. Depending on the form of feeding material, DED can be divided into powder-based DED and wire-based DED. ME is an ‘additive manufacturing process in which material is selectively dispensed through a nozzle or orifice’. MFFF and DMW are two categories of ME. SL represents a 3D printing technique within additive manufacturing that employs sheets of metal as the foundational material. These sheets are successfully adhered to or fused to one another to build layers, which are cut into the desired shape using cutting techniques such as laser cutting. The main SL techniques for metallic materials include UAM and FSAM [31], while laminated object manufacturing is only occasionally used for metals. Table 1 demonstrates the characteristics of DED, ME, and SL. 

SR is critical in manufacturing and engineering applications due to its significant impact on the performance and functionality of components. Ra is a standard industrial measurement for SR and is defined as ‘the mean deviation of the assessed profile’. In Xometry Europe, Ra of 3.2 μm, 1.6 μm, 0.8 μm, and 0.4 μm are four SR levels used for computer numerical control machining applications [60]. Typically, the roughness range for powder-based DED and wire-based DED is Ra 20–50 μm and Ra 200 μm, respectively [61]. Kalami et al. [62] investigated the SR of MAM. The Ra values were 45 μm to 100 μm, 15 μm to 60 μm, 5 μm to 18 μm, and 3 μm to 13 μm for wire-based DED, powder-based DED, PBF, and BJ, respectively. Mao et al. [63] reported that the SR experienced a reduction from Ra of 45.1 μm to 21.9 μm in their LDED study when the oscillating frequency increased from 0 to 90 HZ as the oscillating amplitude was fixed. Singh et al. [64] explored the process control for SR refinement in ME. The initial print results exhibited surface roughness ranging from 6.72 μm to 18.146 μm. By optimizing the layer thickness, nozzle speed, extrusion multiplier, and extrusion temperature, the SR was successfully reduced to 1.6 μm. Based on the above discussion, it is obvious that the reported surface finishes of components fabricated through MAM are poor, and additional surface finishing processes are generally required for industrial applications.

Cost analysis is critical for MAM applications. Kahanna et al. [61] provided a detailed overview of the PBF and DED processes. The relationship between process parameters and overall cost was presented. Dogea et al. [65] conducted a case study of fabricating wing ribs using PBF, and the total estimated cost was USD 1568. Compared to PBF, the costs of DED and ME are significantly less. Table 2 presents cost estimates for DED and ME; the SL case is missing due to the limited cost analyses published for SL. 

In general, SL is desirable for the manufacturing of large products, and the building scales for ME and DED are flexible, while support for overhanging structures may be necessary for ME. Although the mechanism and manufacturing expenses of ME are simpler and more affordable, dimensional inaccuracy due to shrinkage and the need for additional post-processing are two significant limitations. For DED, high residual stress and challenging process control are bottlenecks, while poor dimensional resolution and surface finish are substantial concerns of SL. From the perspective of application, DED is ideal for repairing high-value components. SL exhibits advantages in creating large-scale structures in need of good mechanical properties. ME focuses on fabricating objects at a controllable expense in a safe manner. 

The following sections provide detailed reviews on the mechanisms, material selection, and print quality of non-powder-bed-based MAM techniques. State-of-the-art studies of innovative designs are also reviewed. 

## 3. Direct Energy Deposition

### 3.1. Method and Mechanism

**Figure 2:**
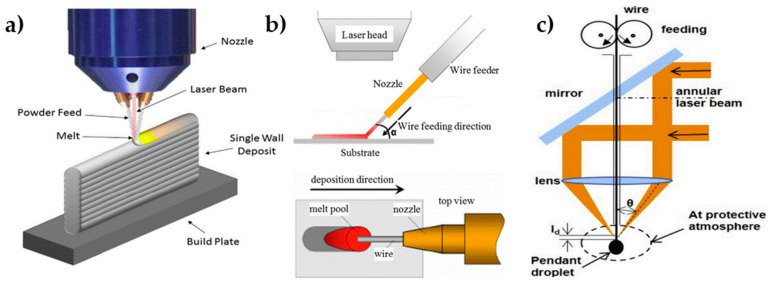
(**a**) Powder-based laser direct energy deposition (LDED); (**b**) LDED with lateral wire feeding; (**c**) LDED with coaxial wire feeding [69,72,73].

Figure 2 demonstrates the mechanisms of three types of LDED. Figure 2a demonstrates the mechanism of LDED using metal powder. Both metal powder and wire feedstocks can be used for LDED. Metal powders are conveyed through a nozzle onto the surface, where a laser beam melts them layer by layer and forms a 3D object. In contrast, LDED using a wire feedstock involves feeding metallic wire onto the substrate and into contact with the laser. The melt pool is formed on the substrate, where the metallic wire is fed and melted, creating a metallurgical bond. A bead is generated by controlling the relative motion between the laser and the substrate after solidification, and the 3D object is then created through continuous processes of feeding, melting, and deposition [68,69,70]. In comparison, the storage of metal wires is safer and more straightforward than that of metal powders. However, melting metal wires usually requires a higher laser power, leading to higher equipment costs for wire-based LDED systems [71]. Figure 2b presents an LDED schematic using a lateral wire-feeding mechanism where the wire feeder transports the metallic wire from the sides. Figure 2c presents a coaxial wire-feeding mechanism, and the metallic wire is fed within the printing device. 

**Figure 3:**
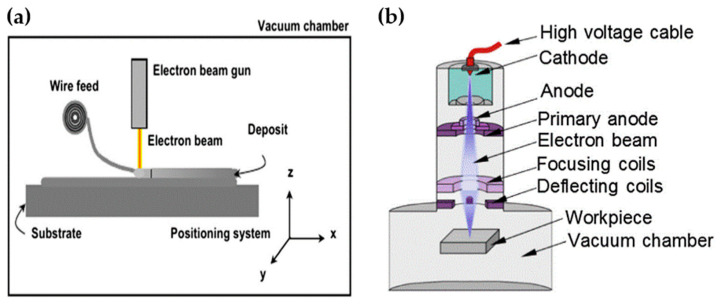
(**a**) Schematic of electron beam direct energy deposition; (**b**) schematic of electron beam device [69,77].

Figure 3a presents the mechanism of EB-DED. In general, EB-DED utilizes a mechanism similar to that of LDED to fabricate 3D geometry. An electron beam gun is used as a heat source, and a vacuum environment is required. A high-vacuum environment is beneficial in creating a less-polluted atmosphere and dealing with reactive metals such as titanium [74,75]. Nevertheless, the vacuum environment may pose a challenge for in situ monitoring, leading to limitations in achieving closed-loop control of the manufacturing process [76]. Figure 3b presents the schematic of an electron beam device or gun, and detailed processes are illustrated in Ref. [77].

**Figure 4:**
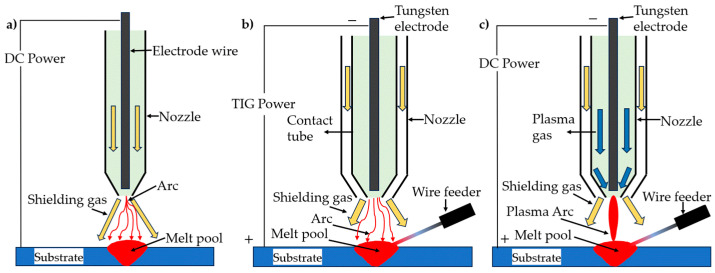
Types of wire-arc additive manufacturing: (**a**) metal inert gas welding; (**b**) tungsten inert gas welding; (**c**) plasma arc welding. Redrawn from Ref. [79].

Figure 4a–c demonstrate three types of WAAM mechanism: MIG, PAW, and TIG. In comparison to common AM processes, MIG stands out as a more user-friendly and convenient approach due to its continuous wire spool with the welding device, while TIG and PAW require additional wire-feeding equipment. MIG utilizes a consumable wire electrode for a high deposition rate but sacrifices precision. On the other hand, TIG welding employs a non-consumable tungsten electrode with an external wire feed. This results in a more complicated process but allows for greater control and precision. PAW also uses a non-consumable tungsten electrode but differs in its more concentrated and intense plasma arc. This intensity provides the finest control over the heat input and weld pool, leading to the highest precision among the three methods [78]. 

**Figure 5:**
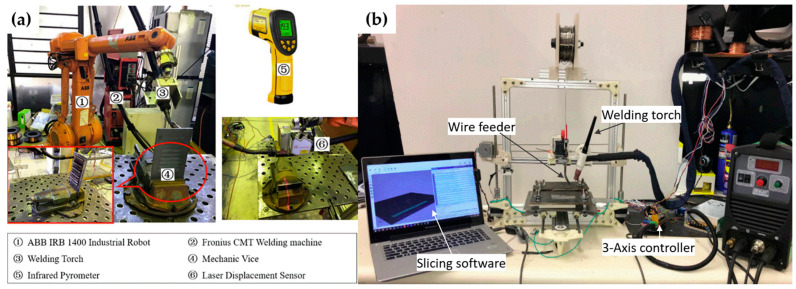
(**a**) Wire-arc additive manufacturing (WAAM) welding torch installed on an ABB robot; (**b**) WAAM welding torch integrated with an XYZ-axis platform [68,80].

Typically, welding torches are installed on a robotic arm to achieve the 3D metal-printing process, as shown in Figure 5a. Due to the high cost of industrial robots and the high flexibility of the WAAM mechanism, researchers have even integrated a welding torch with a portable XYZ moving platform, as shown in Figure 5b.

**Figure 6:**
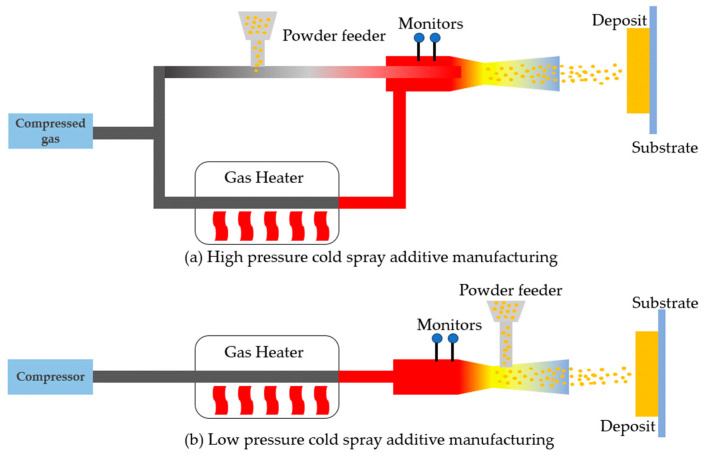
Mechanisms of (**a**) high-pressure cold spray additive manufacturing; (**b**) low-pressure cold spray additive manufacturing (Redrawn from Ref. [26]).

Figure 6 presents the mechanism of CSAM. Depending on the pressure of the propulsive gas flow, the cold spray process can be defined as high-pressure cold spray (>1 MPa) or low-pressure cold spray (<1 MPa). Figure 6a presents a high-pressure CSAM system, where the compressed gas flow passes through a gas heater and works as a propulsive gas; meanwhile, the carrier gas flow passes through the powder feeder channel and carries the metal powder towards the propulsive stream. After mixing two gas flows, it enters the de Laval nozzle, forming a supersonic gas and the powder stream. The pressure of the carrier gas flow should be higher than that of the propulsive gas stream to ensure the success of the powder mixing process. Finally, the accelerated gas and powder stream impacts the substrate, creating a coating or deposit [26]. 

Figure 6b illustrates a low-pressure CSAM system. Instead of compressed gas, a portable gas compressor is usually deployed. The metal powder injection location is close to the nozzle’s divergent section, and metal powder can be released into the stream due to low local gas pressure. In comparison, a low-pressure CSAM system is more affordable and applicable [26,81,82]. 

### 3.2. Materials, Mechanical Properties, and Defects of DED: The State of the Art

There are various material choices for DED. Table 3 presents a variety of material options, elongation, YS, UTS, and reported defects for the four types of DED technologies mentioned. 

Enhancing mechanical properties and preventing printing defects are two key objectives for many researchers. Mao et al. [63] studied the mechanical properties of Al-Cu samples using oscillating laser wire additive manufacturing. The UTS and EL of the fabricated specimens exhibited increases of 40.7% and 20.4% by applying oscillating strategy, respectively; moreover, porosity and cracks were effectively suppressed by applying an 8-shaped oscillating strategy. Liu et al. [87] applied an ultrasonic rolling process to the LDED system and found that defects such as pores were significantly reduced; meanwhile, fine equiaxed grains were achieved. The mechanical properties and microhardness were improved. Wang et al. [88] found that the microhardness of printed samples increased as the laser scanning speed decreased. Moreover, a 1200 ℃ solution heat treatment enhanced the printed samples’ ductility. Pu et al. [95] explored the role of carbon in changing Ni-Ti shape-memory alloys manufactured through EB-DED and found that adding carbon could significantly improve the printed samples’ tensile ductility. A high-carbon Ni-Ti sample could have double the tensile elongation of a low-carbon Ni-Ti sample. Elangovan et al. [109] reviewed the WAAM of aluminum alloys and investigated the causes of defects; prevention strategies were summarized as well. Panicker et al. [110] novelly integrated a rotational arc torch into the WAAM system and reported that the UTS of the printed ER70S6 alloy was increased by 90 MPa compared to previous studies. The rotational arc reduced the net energy input along the bead, leaving less time for grain growth and leading to a finer grain structure [111,112]. Yin et al. [113] reviewed the causes of micropores and interparticle boundaries in CSAM. Increasing the particle impact velocity and applying heat treatment are two methods for reducing defects and reinforcing mechanical properties. Xia et al. [114] studied sensor-based processing, monitoring, and control strategies for WAAM, focusing on identifying and reducing defects. 

### 3.3. Six-Beam Direct-Diode LDED

**Figure 7:**
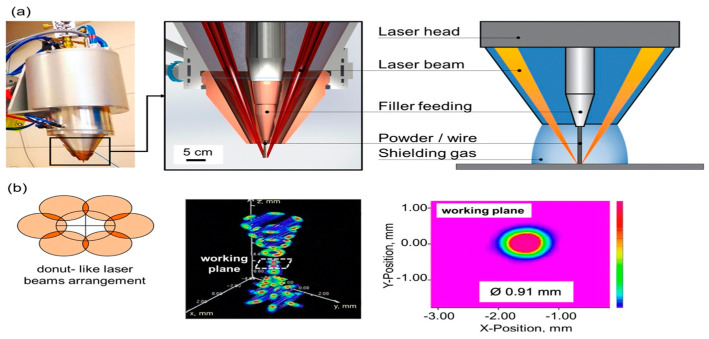
(**a**) LDED head design with coaxial feeding and arrangement of the lens; (**b**) laser beam arrangement demonstration, working plane explanation, and 0.91 mm laser spot measurement [42].

Manufacturing Method: Bambach et al. [42] proposed a six-beam direct-diode LDED apparatus, as shown in Figure 7. The six-beam direct-diode LDED printhead was mounted on an industrial robot, and the maximum laser power was 1 kW. The six-laser beam was generated inside the head and delivered to the substrate and material through a collimator and a focal lens without fibers. An axial mechanism transported the feeding material, and heat resistance provided the preheating for hot-wire printing examination. A highlight of this design is that a more straightforward manufacturing process can be achieved due to the adjustable individual laser beam and coaxial material feeding. Shielding gas protection was ensured compared to conventional LDED systems, where gas stream turbulence causes oxidation accumulation.

Materials and Product Quality: The printing material was the superalloy INCONEL IN718. This material has high creep and fatigue strengths at elevated temperatures. In addition, IN718 exhibits high yield and tensile strength, high ductility, and corrosion resistance. The superalloy IN718 has a variety of applications, such as in gas turbine parts, aerospace components, and nuclear reactors [42]. 

**Figure 8:**
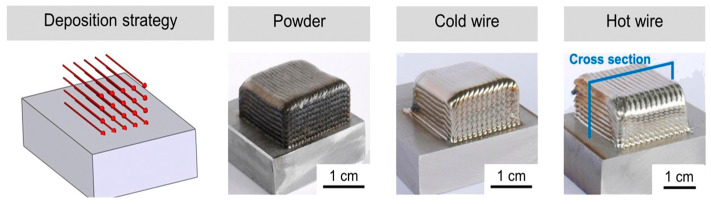
Deposition strategy for printing samples with powder, cold wire, and hot wire [42].

**Figure 9:**
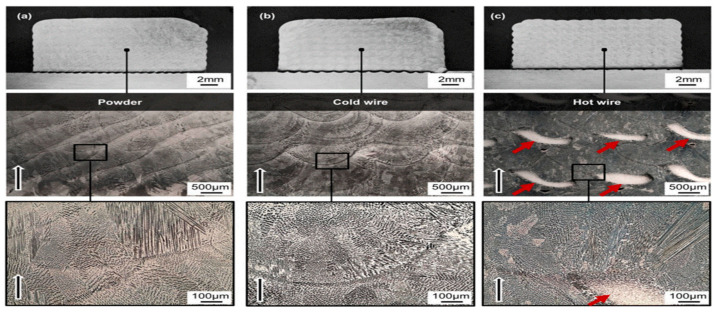
Macroscopic (top) and microscopic (middle, bottom) views of the samples produced using (**a**) powder, (**b**) cold wire, and (**c**) hot wire. The black arrows show the direction of construction. The red arrows highlight the areas of recrystallization in the hot-wire samples [42].

The rectangular cuboid samples are presented in Figure 8. Due to the powder used in the experiment, oxidation occurred in the powder-printed samples. In contrast, cold- and hot-wire-printed samples had shiny surfaces. The cross-sectional images of the three samples exhibit a solid and high-quality print, and the adhesions between single tracks, as well as the substrate material, are free of defects, as shown in Figure 9 [42].

**Figure 10:**
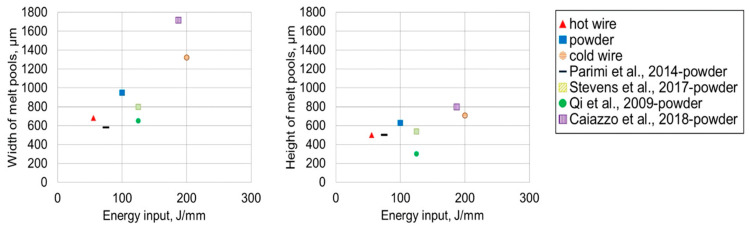
Correlations between the size of the melt pool and the energy input (J/mm) for the hot wire, powder feeding, cold wire, and previous studies [42,43,44,115,116].

According to Figure 10, utilizing hot wire in the fabrication process results in a smaller melt pool size. The dimensions of the melt pool expand as the energy input increases, as proven by previous researchers [53,54]. Preheated filaments require less printing energy than cold wires or powders, making it more energy-efficient to complete the AM process more precisely. Therefore, preheating the filament reduces the melt pool dimensions and enhances the printing resolution [42].

Summary: Overall, a six-beam direct-diode LDED using an adjustable individual laser with gas shielding is feasible for printing high-quality materials. The preheating of the feeding wire can successfully reduce the melt pool size and improve the print resolution. However, this approach achieves substrate adhesion by melting and fusing the substrate, and this mechanism can lead to additional post-processing to remove printed objects from the substrate.

### 3.4. Liquid-Metal-Assisted DED

**Figure 11:**
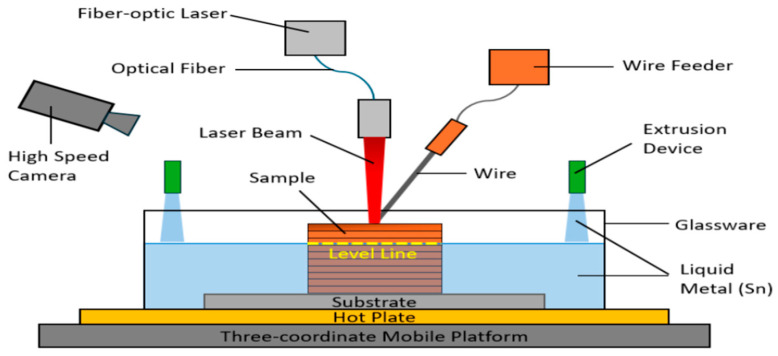
Liquid-metal-assisted direct energy deposition mechanism (redrawn from Ref. [126]).

Manufacturing Method: As shown in Figure 11, liquid-metal-assisted DED ingeniously incorporates liquid tin as a thermal management material for DED. Compared with previous parameter optimization research [117,118], deposition element composition adjustment [119,120,121,122], beam shape control [123,124], and integrated auxiliary methods [125], this novel approach effectively overcomes the limitations faced by conventional thermal management techniques, such as limited thermal management zones, thermal profile response rates, and high equipment expenses [126].

A key highlight of the liquid-metal-assisted DED process is enhanced real-time temperature and stress control during metal deposition. This capability is crucial for ensuring the structural integrity of the final product. The incorporation of liquid tin yields significant improvements. This mechanism effectively transfers heat between the sample and the liquid tin, decreasing the sample’s peak temperature.

**Figure 12:**
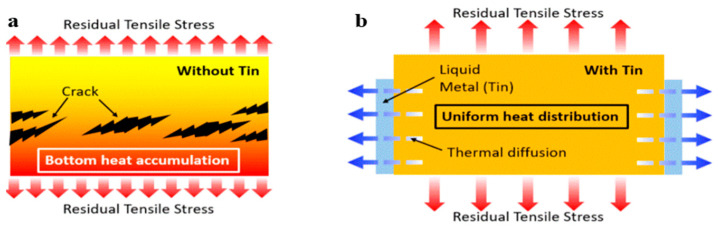
(**a**) Residual stress and defects without liquid tin’s assistance; (**b**) residual stress with liquid tin’s assistance (redrawn from Ref. [126]).

By controlling the level of liquid tin, the melt pool size and the peak temperature can be adjusted accordingly. Moreover, liquid tin contributes to thermal diffusion in the printing process, providing a sample with a uniform temperature distribution, coordinated deformation and, eventually, appropriate residual stress management, as shown in Figure 12 [126].

Materials and Product Quality: The liquid-metal-assisted DED process uses a 1.2 mm long Ti6Al4V wire as the feedstock. Ti6Al4V is regarded as the most popular titanium alloy and has a variety of applications in different industries, e.g., the aerospace industry [127,128] and biomedical industry [129].

Tin was selected as the auxiliary thermal management material because of its excellent thermal conductivity and fluidity in its liquid state. An inert coating was applied to Ti6Al4V to avoid chemical reactions between the feedstock and tin. Ti6Al4V sheets were utilized as the printing substrate.

**Figure 13:**
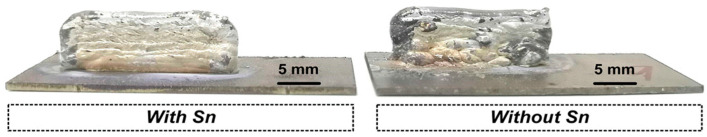
Printed samples with and without tin’s assistance [126].

Figure 13 shows the effectiveness of liquid tin, where the sample with tin assistance presented a slightly higher print quality than the sample without tin, and the warpage was less than that of the sample without tin. The cooling rate increased by approximately 20%, and the peak temperature decreased by approximately 400 ℃ due to heat transfer between the liquid tin and the deposited material. By adjusting the liquid tin level, the non-uniform heat distribution caused by heat accumulation was limited, leading to a 30% reduction in the residual stress and geometrical deformation [126].

**Figure 14:**
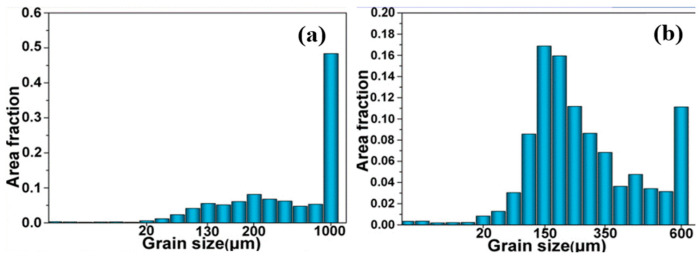
Grain size distribution for (**a**) without tin and (**b**) with tin [126].

As shown in Figure 14, the application of tin leads to finer grain sizes in the deposits (approximately 150 µm, compared to 200 µm without liquid tin’s assistance), characterized by alternating basket and lamellar structures and devoid of visible cracks. This is due to the increased cooling rate after tin is involved, as a higher cooling rate offers more distortion energy for grain refinement [130]. Finer grains in the microstructure also contribute to an approximately 30% higher microhardness, which corresponds to the Hall–Petch theory [126,131].

Summary: On the one hand, this study successfully validated the effectiveness of liquid tin as a thermal management medium. The sample with liquid tin’s assistance presented better geometry, and the warpage at the corner was restricted. In addition, liquid tin contributed to finer grain formation and higher microhardness. On the other hand, the quality of the fabricated sample was not as high as desired, and the sample had a poor surface finish. Therefore, a detailed machining process is required.

### 3.5. Hot-Forge WAAM 

Manufacturing Method: Hot-forge WAAM innovatively combines WAAM with hot forging, where the material undergoes in situ viscoelastic deformation at high temperatures immediately after deposition [132]. Compared with previous research aimed at minimizing porosity and refining grain size, such as cold rolling [133] and hot rolling [134,135], there are no significant additional equipment updates, and the WAAM’s efficiency can be improved while simultaneously ensuring mechanical property enhancements and microstructure refinement [136].

**Figure 15:**
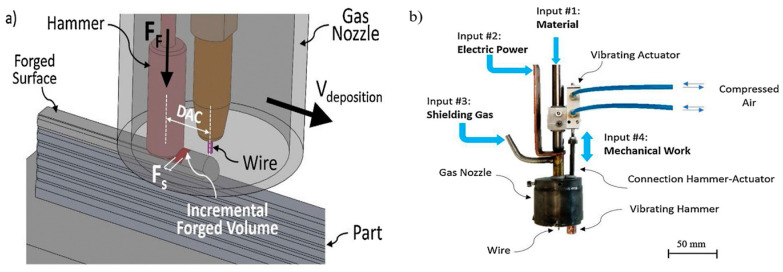
(**a**) Mechanism of hot-forge WAAM; (**b**) hot-forge WAAM printing device [136].

Figure 15 shows that a vibrating actuator-driven hammer was mounted inside the gas shield and could work at various frequencies. While traveling along the torch, the hammer impacted the layer deposited at a high temperature. The hammer was connected to the power supply through a high-resistance connection, which limits the current flow when it contacted the fabricated sample. This customized WAAM torch was mounted on a moving head with a preset working zone. A PRO MIG 3200 W power source was utilized to print the object with 1 mm diameter AISI316L stainless steel on a mild steel substrate. The forging temperature in this process was around 900 ℃, exceeding the recrystallization temperature of AISI316L stainless steel [136].

Materials and Product Quality: AISI316L stainless steel was used as the print material, with a recrystallization temperature of around 450 ℃. AISI316L stainless steel is a common material for MAM. Danilo et al. [137] reviewed the application of AISI316L stainless steel in additive manufacturing using various MAM techniques. Majumdar et al. [138] studied the application of AISI316L stainless steel in bio-implants. Pradeep et al. [139] explored the application of AISI316L stainless steel in aerospace applications. 

**Figure 16:**
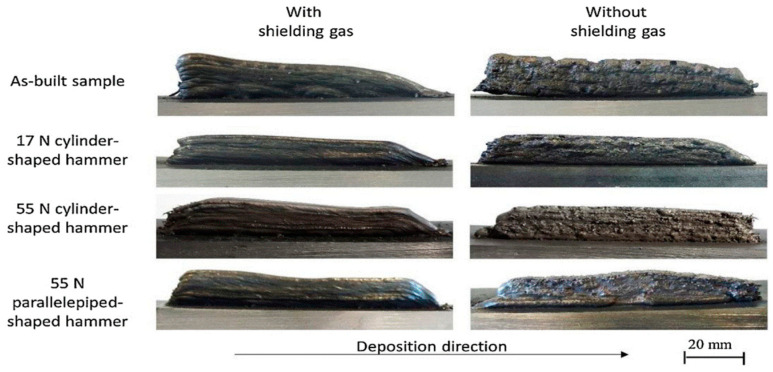
Demonstration of samples printed with different process parameters [136].

Figure 16 depicts samples printed with different hammer forging forces, hammer shapes, and shield gas flows. Samples without gas shielding exhibited more pores, and warpages were observed between the substrate and the first track when the hammer force was not applied. In comparison, samples with gas shields presented better surface finishes.

**Figure 17:**
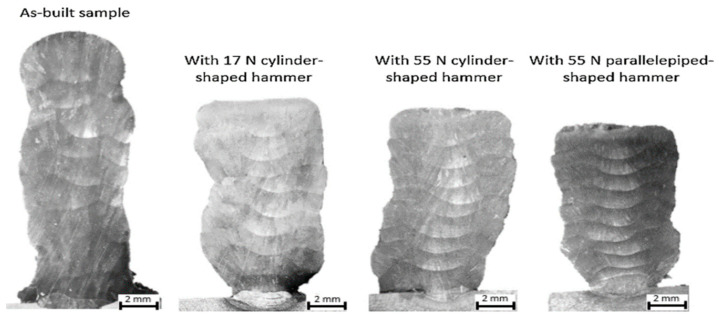
Cross-sectional view of samples fabricated with shield gas and different hammer forces [136].

**Figure 18:**
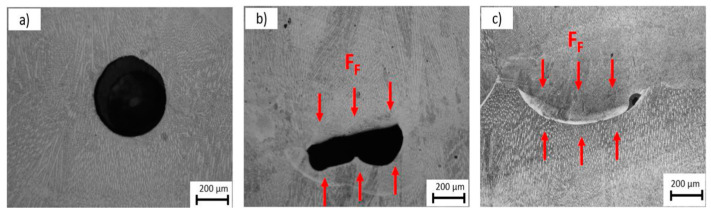
Porosity assessment for (a) 17 N forging, (**b**) 55 N forging with a cylindrical hammer, and (**c**) 55 N forging with a parallelepiped-shaped hammer [136].

Figure 17 demonstrates the effects of hot forging. The layers were larger and thinner when the forging force increased, and this phenomenon became more apparent when a rectangular hammer with a smaller contact area was utilized. Moreover, the hot forging process resulted in a flatter deposition, which was beneficial for printing new layers. As shown in Figure 18, the number of pores decreased during the hot forging process with increasing forging force. At the same time, a parallelepiped hammer, which offered a reduced contact area, had an even more significant effect on pore collapse. For the microstructure evolution aspect, more nucleation sites were present due to the hot forging process, reducing the grain size in the layer. The UTS of the hot-forged sample was approximately 8.6% greater than that of the as-built sample, and a decrease in ductility was observed, from 32.5% for the as-built sample to 27.5% for the same sample with hot forging applied [136].

Summary: This research focused on reducing porosity in printed samples, and a hot forging process can diminish pores. In addition, the mechanical strength increased due to the hot forging process. On the other hand, although the concept was validated, the quality of the printed samples could be improved in future studies.

## 4. Material Extrusion

### 4.1. Method and Mechanism

**Figure 19:**
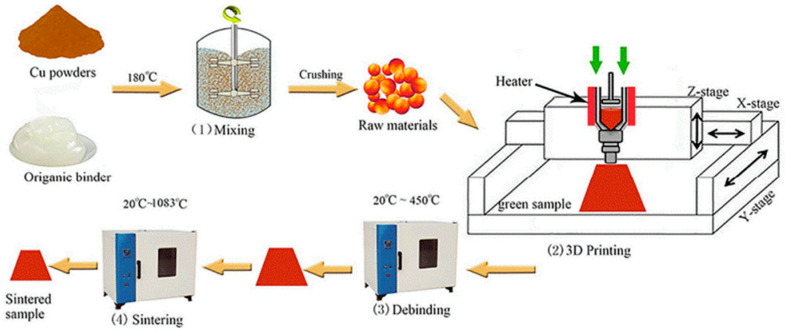
Schematic of metal fused-filament fabrication (MFFF) process [145].

Figure 19 illustrates the schematic of the MFFF process. As the first step, feedstock preparation is of fundamental significance in MFFF. Selecting suitable metal powders and binder systems is a key mission in this step. The criteria of metal powder selection are as follows: (1) a small size of metallic particle is desired, (2) the powder needs to have a good dispersion in the binder system, (3) a sintering process is required for densification, and (4) the sintering and melting temperatures should be higher to avoid interference with the debinding process [27,140]. Particularly, a smaller size of metallic particle is preferred because larger powder particles can lead to poorer printability and even print failure [141,142,143]. Metallic powder loading is typically 55% to 65% by volume to achieve densely sintered components, while a higher metal powder filling can lead to lower printability [144]. 

Generally, a backbone binder, a second polymeric phase, and other additives are the three types of materials that form the binder system. The backbone binder is responsible for maintaining the component’s structural integrity after the debinding process, while the second polymeric phase is utilized to adjust the feedstock’s viscosity, which can be easily removed at low temperatures. Additives play an essential role in achieving excellent diffusion between the metal powder and the binder, preventing phase separation and agglomeration [27,146]. 

Researchers have explored combinations of binder systems. Ren et al. [145] combined paraffin wax, low-density polyethylene, and stearic acid in their binder system. More studies of binder system combinations are reviewed in Ref. [27]. 

**Figure 20:**
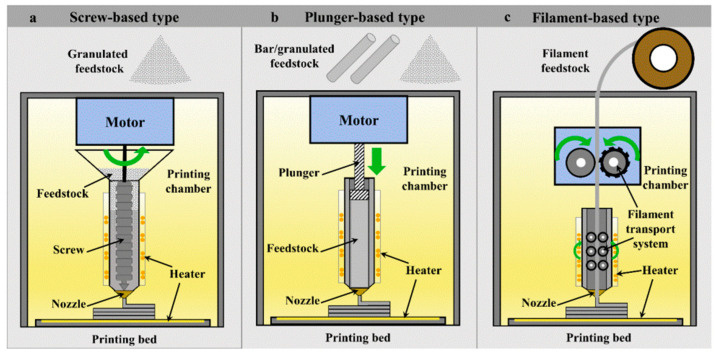
Different types of MFFF processes classified by material feeding mechanisms: (**a**) screw-based; (**b**) plunger-based; (**c**) filament-based [147].

In the MFFF process, the feedstock is heated inside the nozzle until it becomes viscous enough to be extruded from the nozzle’s orifice. Then, the extruded material is deposited onto a heated substrate, and the 3D component (green part) is fabricated through a layer-by-layer mechanism. Figure 20 demonstrates three types of MFFF systems. 

Accurate control of the printing process is a key challenge in MFFF. To address this issue, Riaz et al. [148] proposed a combination of polyMIM 8740 and screw-based composite extrusion modeling technology, where an innovative EM-Tower strategy was applied to identify the optimal extrusion multiplier value for precise flow rate control. The extrusion rate control is shown in the following equation based on the relationship between EM and filament diameter ($d_{f}$) [148]:

(1) $$E=\frac{h\cdot d}{\frac{\pi }{4}{d_{f}}^{2}}\cdot l\cdot EM$$

where E is the filament length, h is the layer thickness, b is the oriented line width, and l is the print length of one extruded line. In the composite extrusion modeling 3D printing process, the production of green parts is affected by five key printing parameters: *EM*, extrusion temperature, layer thickness, nozzle velocity, and nozzle diameter. The EM governs the material’s flow rate, and the modification of *EM* and extrusion temperature primarily regulates the viscosity and the ratio of the volume flow of the material, respectively [148].

**Figure 21:**
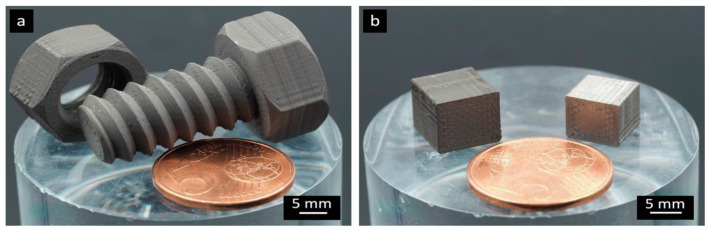
Sample images of MFFF for polyMIM 8740 steel pellets: (**a**) ‘green parts’ of the screw and nut; (**b**) ‘green part’ cubes before sintering (left) and after sintering (right) [148].

Based on an optimization study of the effects of processing parameters on surface roughness, the optimization of parameters—*EM* at 107.6%, extrusion temperature at 180 °C, nozzle velocity at 20 mm/s, and layer thickness at 0.050 mm—demonstrates a solid understanding of the MFFF process. Parts fabricated under these printing parameters are shown in Figure 21.

**Figure 22:**
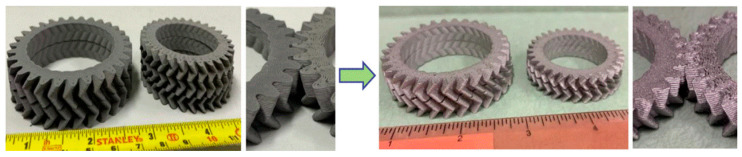
Demonstration of ‘green parts’ (**left**) and ‘sintered parts’ (**right**). Debinding in acetone for 3 h and sintering for around 8 h [149].

Once the green part is manufactured, it will be subjected to debinding and sintering processes to get rid of the binder system and create a fully metallic component, and shrinkage occurs in this phase. Figure 22 presents the fabricated ‘green part’ and the corresponding ‘sintered part’. The green part has a density of 4.98 g/cm^3^, and the shrinkage is 14% after the debinding and sintering processes [149]. 

**Figure 23:**
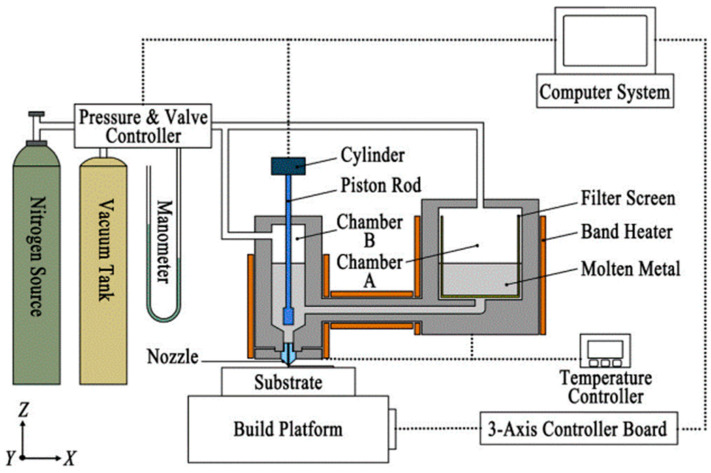
Schematic of pneumatic extruding direct metal writing (DMW) [150].

Compared to MFFF, debinding and sintering post-processes are unnecessary or less complicated in DMW. Ma et al. [150] developed a pneumatic extruding DMW system, as shown in Figure 23. The molten Sn63Pb37 alloy is extruded from the nozzle orifice through the cylinder piston rod and nitrogen purge. 

**Figure 24:**
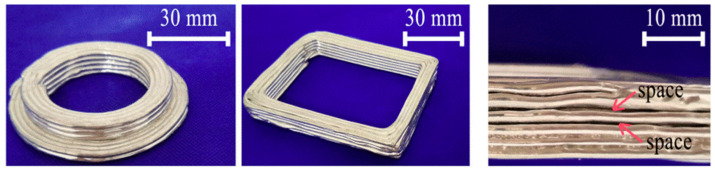
Printed sample using pneumatic extruding DMW [150].

Figure 24 presents the printing results of pneumatic extruding DMW. Print non-uniformity and inter-layer adhesion are two critical limitations among these samples. Print uniformity is crucial for high-quality metal printing, and maintaining a constant molten metal flow rate is essential for achieving a uniform print. Any nozzle clogging or blockage during the printing process can lead to an inconsistent molten metal flow rate and eventually lead to compromised print resolution or even print failure [151]. Poor inter-layer adhesion can be explained by insufficient inter-layer temperature, since the higher layers are further away from the heated substrate. Chen et al. [152] studied the inter-layer adhesion mechanism and stated that the inter-layer temperature should be within the semisolid range to form metallurgical solid bonds. 

**Figure 25:**
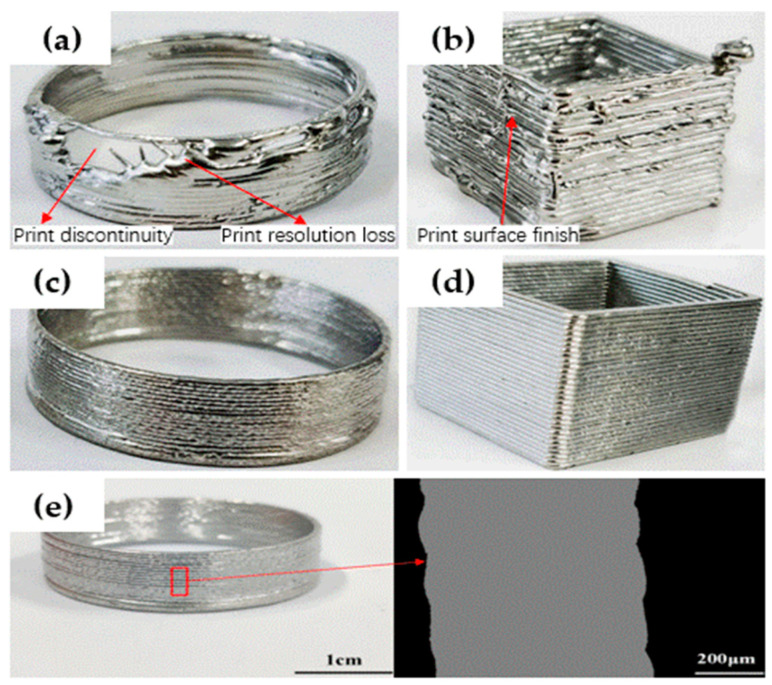
Print quality of samples using (**a**,**b**) DMW (**c**,**d**) ultrasound-assisted DMW, and (**e**) Interlayer bonding of the highlighted area [151].

In addition to the poor inter-layer bonding, print quality is another limitation of DMW. Figure 25a,b highlight the print quality of DMW samples. Low print resolution is a significant drawback of DMW, related to molten metal’s characteristic low viscosity and high surface tension. The extruded molten metal exhibits low viscosity, usually a few hundred centipoises; meanwhile, the ultrahigh surface tension leads to print instability, such as coalescence and print discontinuity. Therefore, the print quality of DMW is significantly compromised [153]. Additionally, the pneumatic extrusion technique, which utilizes pure molten metal as an ‘ink’, is hindered by subpar forming performance, largely due to difficulties in precisely controlling the flow rate at the orifice [152,154].

In response to these challenges, Hu et al. [151] proposed ultrasound-assisted DMW. This novel technique combines the extrusion effect of fed metal wire with the crushing impact of ultrasonic vibrations on molten slag. The apparatus uses a commercial FFF printer with an adjusted preload and an all-metal double-gear extrusion module, a specially designed electromagnetic induction coil, and a piezoelectric ultrasonic transducer. This method effectively mitigates the clogging issues caused by the adsorption and aggregation of molten slag, a common problem in previous techniques. The ultrasonic vibration facilitates the extrusion process, resulting in a smooth extrusion process and a continuous powder flow rate [155,156]. Furthermore, the ultrasound-assisted approach allows for real-time control of the metal flow rate at the orifice, ensuring smooth, continuous writing and significantly improving the forming quality of 3D metal parts. The effectiveness of ultrasonication is demonstrated in Figure 25c,d. Full layer adhesion was achieved in the printed sample due to sufficient heat accumulation during the process. Figure 25e presents the fabricated hollow cylinder with a fully fused cross-sectional view. The substrate adhesion and inter-layer bonding mechanism are critical in achieving high-quality printing.

### 4.2. Materials, Mechanical Properties, and Defects of ME: The State of the Art

The choice of materials for MFFF is diverse, as reported in Table 4, although there have been limited studies on DMW. Moreover, most research has focused on low-melting-point alloys, such as bismuth, tin, or indium alloys. 

**Figure 26:**
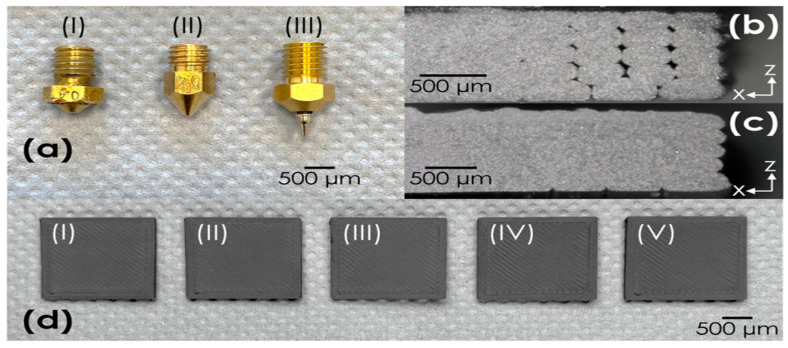
(**a**) The examination of nozzle designs with a 0.4 mm diameter; (**b**) a microscopic view of a sample printed at speeds of 6 mm/s (first layer) and 12.27 mm/s (second layer); (**c**) a microscope image of a sample produced at 4 mm/s (first layer) and 8.18 mm/s (second layer); (**d**) samples created to evaluate reproducibility using speeds of 4 mm/s (first layer) and 8.18 mm/s (second layer); (I–V) represent the number of printed samples [168].

Porosity and voids are common defects that are frequently mentioned in literature. The existence of porosity and voids can significantly compromise the mechanical performance of MFFF builds. Olef et al. [164] reviewed the process monitoring for ME. Temperature, vibration, acoustic emission, electrical quantities, force and pressure, and other sensor monitoring strategies were investigated. Mousapour et al. [48] studied the relationship between debinding rates and porosity levels, finding that a lower debinding rate led to less porosity. Singh et al. [165] found that a sufficient amount of binder is necessary on the surface of the filament to prevent voids during deposition; meanwhile, controlling overlapping is critical for achieving a more condensed component. Wang et al. [166] used HIP after the MFFF process and found that the porosity percentage decreased from 7.5% to 0.3%. Malagutti et al. [167] introduced a remelting and compaction strategy in post-processing and found a remarkable enhancement of density and mechanical properties in post-processed samples. Waalkes et al. [168] developed a piston-based mechanism and studied the correlation between print speeds and voids. Figure 26b presents a microscopic view of a sample printed at speeds of 6 mm/s (first layer) and 12.27 mm/s (second layer), where apparent voids were observed. Figure 26c demonstrates a microscopic view of a sample printed at speeds of 4 mm/s (first layer) and 8.18 mm/s (second layer), where the voids disappeared. This highlights the impact of print velocity on void formation during printing. Figure 26d showcases five printed samples using the same printing parameters as in Figure 26c, and reproducibility was confirmed. 

### 4.3. Bio-Based MFFF

**Figure 27:**
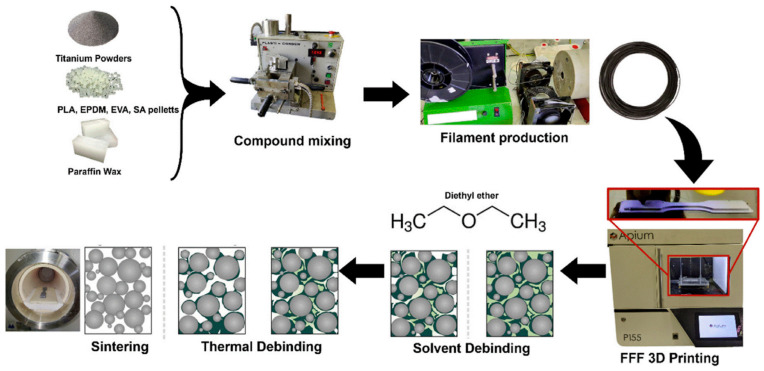
Schematic of the manufacturing process of bio-based MFFF [146].

Manufacturing Method: Inspired by the emerging concerns about the costs of powder-bed-based metal additive manufacturing and the environmental friendliness of PLA, Bragaglia et al. [146] conducted a study that combined MFFF with bio-based Ti6Al4V-filled filaments. Figure 27 demonstrates the workflow of bio-based MFFF. 

Instead of using commercialized feedstocks such as those described in the previous sections or previous studies [169], Bragaglia et al. [68] fabricated bio-based filaments through the compound mixing process, with strict process control. The feedstock was manufactured in a closed-chamber batch mixer for 30 min at 180 °C, with a rotation speed of 20 rpm. Then, it was mechanically ground and dried in an oven for 24 h at 50 °C. 

Materials and Product Quality: The feedstock used in this study consisted of titanium powder as the metal filler and PLA as the backbone binder. It contained 55% *v*/*v* titanium powder and 45% *v*/*v* binder. Using the same printing mechanism, Jiang et al. [170] fabricated biodegradable composite scaffolds using a combination of PLA and 316L stainless steel. Buj-Corral et al. [171] explored the characterization of biomedical steel-filled PLA composite scaffolds. Ali et al. [172] reviewed schematics of the MFFF process for polymer/metal scaffolds, with a detailed study of Mg-based composites. 

The filaments in Bragaglia’s study were prepared using a single-screw extruder (FILABOT EX2, Filabot, Barre, VT, USA) with a nozzle diameter of 1.75 mm. The preparation was conducted at 180 °C, with an extrusion speed of 15 rpm. To enhance the adhesion of the first layer, a polyvinylpyrrolidone-based fixative, known as Dimafix, was applied to the glass bed of a 3D printer. The Apium printer was used to fabricate 3D samples; tensile test samples were printed according to the ASTM standard. 

**Figure 28:**
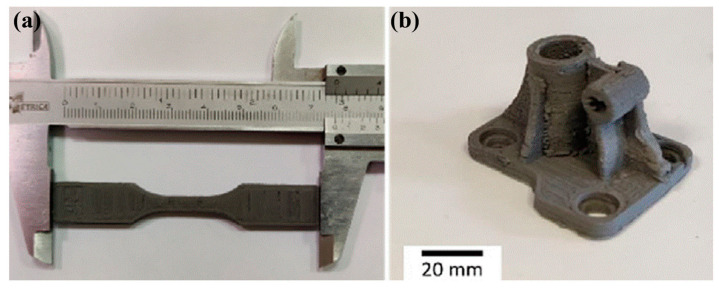
(**a**) ASTM standard tensile test specimen; (**b**) ribbed scaffolding base plate [146].

A ribbed scaffolding base plate was also fabricated as a proof of concept and application, as shown in Figure 28 [146].

The tensile strength of the tensile test specimen was 662.4 ± 12.2 MPa, significantly lower than that of traditional manufacturing processes and less than that of PBF and DED [173,174,175]. However, mechanical property improvements were still made in this research compared to the previous study by Thompson et al. [176], which reported a tensile strength of 350 MPa.

Summary: This section presents the mechanism for bio-based MFFF, where biodegradable PLA is utilized as a backbone binder. In general, bio-based MFFF is an innovative approach to creating biodegradable components. The complexity of the printing system is less compared to other MAM processes. In addition, the printing quality and resolution of the samples are more satisfactory, and there are various material choices for this process. Nevertheless, bio-based MFFF still requires a series of processes and post-processes, such as binder-filled metal feedstock manufacturing, debinding, and sintering processes. Shrinkage is one of the significant issues in MFFF and can cause dimensional inaccuracies and printing resolution losses.

### 4.4. Thixotropic ME

**Figure 29:**
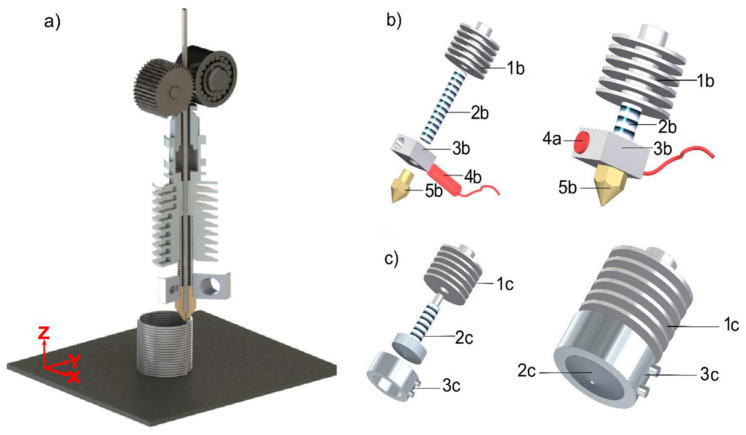
(**a**) Commercial FFF mechanism; (**b**) commercial FFF-printed hot end; (**c**) design of the thixotropic hot end [179].

Manufacturing Method 1: Recently, many researchers have focused on filament-based thixotropic ME. Zhang et al. [177] presented a thixotropic ME system using a Pb-Sn filament. Using a similar mechanism, Nezic et al. [178] used an Al4018 aluminum alloy filament to print objects manually. In addition, the choice of nozzle inlays was explored. Copper and stainless steel inlays were studied to find a suitable inlay material for a smooth filament extrusion process, and copper inlays were proven to be effective for reducing extrusion friction. Figure 29 presents a thixotropic ME mechanism proposed by Lima et al. [179]. The thixotropic ME system in Lima’s work is based on a Prusa FFF printer with a specially designed extrusion system.

Materials and Product Quality 1: A biodegradable zinc–magnesium alloy composition strategy (liquid fraction, liquid fraction sensitivity, etc.) was used. Mg-38Zn was chosen as the experimental candidate following thermodynamic simulations that pinpointed promising alloys for FFF. This selection process was underpinned by thermodynamic simulations and differential scanning calorimetry analysis. These techniques were instrumental in examining the alloy’s transition from solid to liquid, with differential scanning calorimetry results corroborating the simulations and verifying the aptness of the chosen composition [179]. Notably, a similar composition selection strategy was used to select a suitable Sn-Bi alloy for thixotropic printing in Lima’s recent research [180]. Using a similar mechanism, Nezic et al. [178] and Zhang et al. [177] applied Al-Si and Pb-Sn alloys in filament-based thixotropic ME. 

For filament preparation in Lima’s study, the as-cast Mg-38Zn alloy and Sn-38Bi alloy ingots underwent a hot extrusion process with a hydraulic pressing mechanism. Mg-Zn alloys are well known for their excellent biodegradability, and they are widely used in the medical industry due to their biocompatibility, corrosion resistance, and high strength. Both elements have benefits to the human body, such as human bone growth. Mg alloys can even minimize ‘stress shield phenomenon’ since the elastic modules of Mg alloys and human bone are similar [181,182]. Sn-38Bi alloys are mainly used in the microelectronics industry because of their low melting point, good wettability, and high strength during jointing [183].

For the Sn-38Bi alloy, a significant achievement was successful thixotropic meal printing at 170 °C, where the alloy was extruded and deposited without any nozzle clogging or discontinuities. Moreover, nozzle clogging and discontinuities were recurrent at 140 °C, and a higher extrusion force was needed at 152 °C, where the liquid fraction was 0.5 to 0.8 for a temperature window between 140 °C and 170 °C [180]. For the Mg-38Zn alloy, the semisolid Mg-38Zn exhibited the best rheological behavior for the printing process at 420 °C. Temperature, shear rate, time, and solid-phase morphology are the four main factors determining semisolids’ rheological behavior [132]. In addition, a globular solid-phase morphology is a prerequisite and can be achieved with appropriate heat transfer and shear [179].

**Figure 30:**
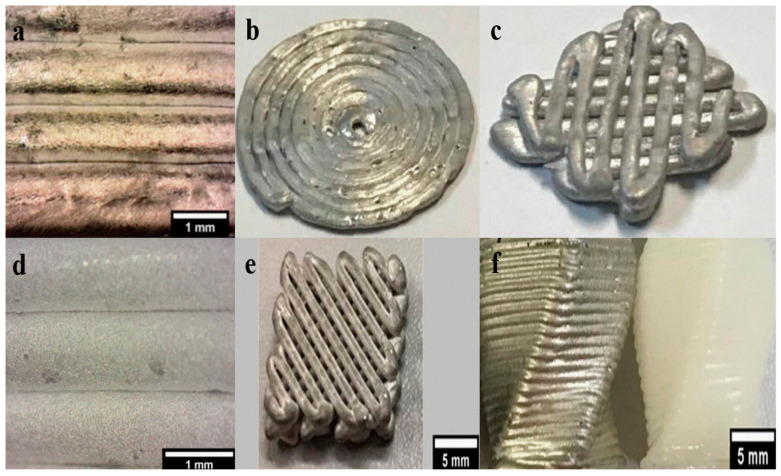
Printed Mg-38Zn semisolids: (**a**) multilayer structure; (**b**) spiral structure; (**c**) two-layer structure. Printed Sn-38Bi semisolids: (**d**) multilayer structure; (**e**) two-layer object; (**f**) printed component (Sn-38Bi) (left) and printed component (polylactic acid) (right) [179,180].

Figure 30a presents a detailed view of the filaments’ contact points, where no geometrical loss was observed. Figure 30b demonstrates a printed spiral surface. Although the overall shape of the spiral surface was maintained, the inter-filament adhesion was not consistent. Figure 30c depicts a two-layer structure, where the print quality was limited and inter-layer fusion was not ensured. Figure 30d–f show the views of printed objects using Sn-38Bi alloy, and the print quality was improved compared to previous results. 

Summary 1: Lima’s study underscores the practicality and advantages of using a Mg-38Zn alloy and Sn-38Bi in a semisolid state for AM. This highlights the beneficial microstructural characteristics and fabrication potential of these alloys, thereby contributing significantly to the field of additive manufacturing. The inter-layer and substrate adhesion mechanisms could be explored in detail in further studies. Other materials, such as Pb-Sn and Al4018 alloys, also hold the potential for thixotropic ME. 

**Figure 31:**
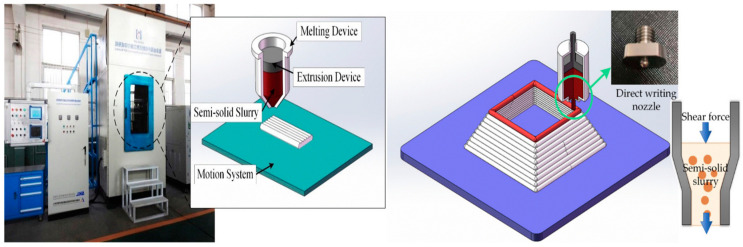
Semisolid direct-writing system [184].

Manufacturing Method 2: Rong et al. [184] presented a different approach using piston-based thixotropic ME, combining semisolid formation with ME. The semisolid direct-writing tests were conducted in a semisolid aluminum melting direct-writing apparatus, which contained a melting system for heat treatment, an extrusion system, and a 3D motion system [184], as shown in Figure 31.

Materials and Product Quality 2: The semisolid isothermal heat treatment involved a three-step process. During the heat treatment, the cast aluminum billet was deformed above the recrystallization temperature and then cooled to room temperature. The recrystallized billets were directly heated to a semisolid state, and the morphology evolved from elongated crystals to equiaxed crystals to spherulites [184,185].

**Figure 32:**
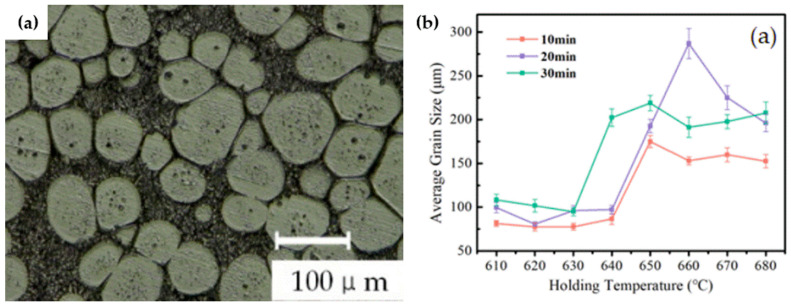
(**a**) Thixotropic microstructure of 2A12 alloy held at 650 °C for 25 min; (**b**) correlation among average grain size, holding temperature, and holding duration for 2A12 alloy [184].

One of the standout achievements in Rong’s study is the identification of optimal conditions for this process: a holding temperature of 640–650 °C and a holding time of 20–25 min, achieving a liquid-phase rate of approximately 40%, with an average grain size of 150 μm. Figure 32a presents a thixotropic microstructure of 2A12 alloy held at 650 °C for 25 min. Figure 32b demonstrates the correlation among average grain size, holding temperature, and holding duration, where a higher holding temperature and longer heating duration are more likely to create larger grains [184].

**Figure 33:**
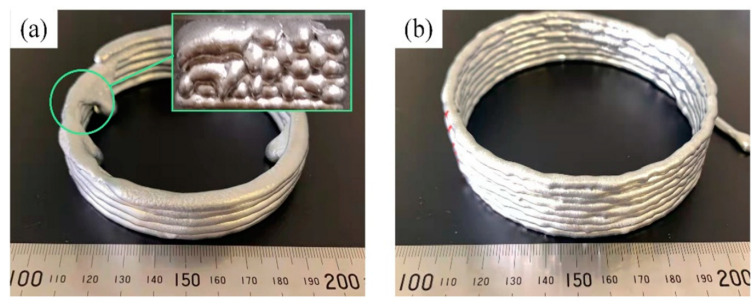
Semisolid melt direct-writing specimens with different liquid-phase volume fractions: (**a**) liquid melting direct-writing sample; (**b**) semisolid melt direct-writing sample with 50% liquid fraction during writing [184].

The isothermal semisolid heat-treated material was then applied to the extrusion system. The piston can provide an extrusion force between 0 and 10 KN and offer a uniform shear force for the semisolid slurry to be pushed out of the printing nozzle. The deformation resistance decreases when shear is applied, making the semisolid slurry flow like a liquid and forming an object layer by layer, as shown in Figure 33 [184,186].

As shown in Figure 33b, the semisolid slurry supported the printed structure after being deposited on the substrate, and the structure did not deform during the printing process. Based on the experimental data, a liquid fraction ranging from 35% to 50% is needed for continuous and uniform deposition processes.

Summary 2: Rong’s study presents a feasibility study of a thixotropic ME using a preheated billet. A globular grain structure was created through a hot work and reheating process. The 2A12 aluminum alloy is a high-strength metal alloy that has ahigh strength-to-weight ratio. Applications of this alloy include aircraft structural components and automobile parts. However, few samples were demonstrated in this study, and the mechanism of thixotropic 3D metal printing could be developed in subsequent exploration.

Manufacturing Method 3: Inductive thixotropic ME utilizes an induction heater to heat the metal alloy to a semisolid state, and the semi-solid metal will be extruded out of nozzle through continuous material feeding. Englert et al. [187] utilized an IH mechanism in their customized thixotropic ME apparatus, and an IH power reduction strategy was applied to print a cubic aluminum structure. Sharma et al. [188] used direct IH for thixotropic ME to improve the processing efficiency. 

**Figure 34:**
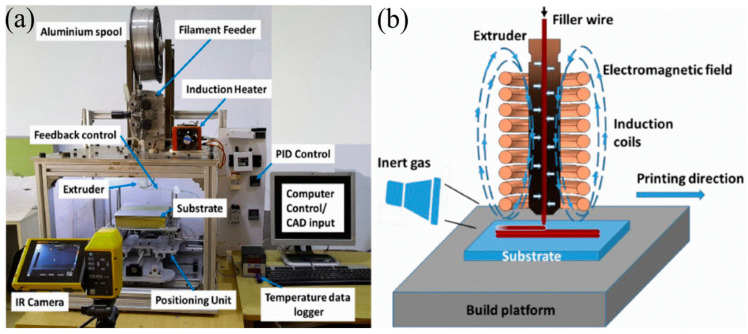
(**a**) Thixotropic material extrusion prototype using an induction heater; (**b**) schematic of inductive thixotropic ME [188].

As shown in Figure 34, the apparatus contains an extrusion module that integrates the functions of wire feeding, material extrusion, IH with a coil management and cooling system, and an X-Y-Z-axis motion platform. A proportional integral derivative-based temperature controller controls the print temperature, and computer-aided design software controls the motion.

In addition, an in situ infrared camera was used to investigate the thermal field evolution and detailed thermal profile to determine the temperature distribution during printing. Inert gas was purged from the side during printing, preventing oxidation from occurring [188].

Materials and Product Quality 3: For this study, a 1.6 mm diameter filament of Al-5356 alloy was utilized as the printing material, and an Al-5000 series aluminum alloy was selected as the substrate material. Al5356 alloys are widely used and applied in industry due to their superior strength-to-weight ratio, exceptional ductility, ease of welding and forming, and resistance to corrosion [189,190].

**Figure 35:**
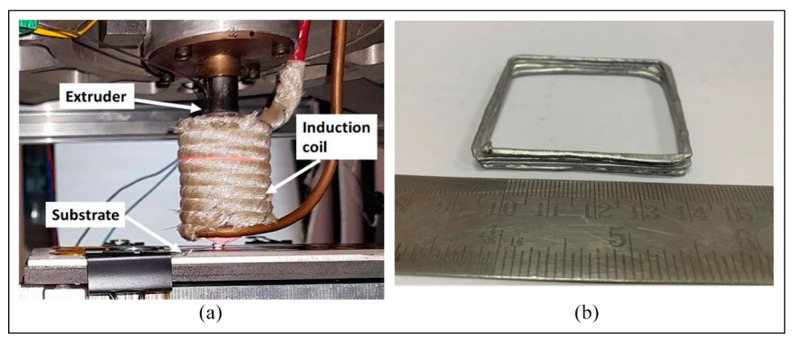
(**a**) Printing shortcut during inductive semisolid metal manufacturing; (**b**) a multilayer rectangular part [188].

This study also investigated the effects of substrate position and heating on the process, concluding with promising future applications for improving the quality of materials deposited in printing. Figure 35a shows a macro-image of the printing process, with the extrusion head close to the substrate, and Figure 35b shows a four-layer structure.

Summary 3: In general, Sharma presented a unique approach for thixotropic ME by applying IH, showing that IH is an emerging, cost-effective, clean, safe, and precise energy source. On the other hand, demonstrations of printed samples are insufficient, and analyses of sample quality are lacking. In addition, the temperature control of IH needs to be improved for better printing process control.

## 5. Sheet Lamination

### 5.1. Method and Mechanism

**Figure 36:**
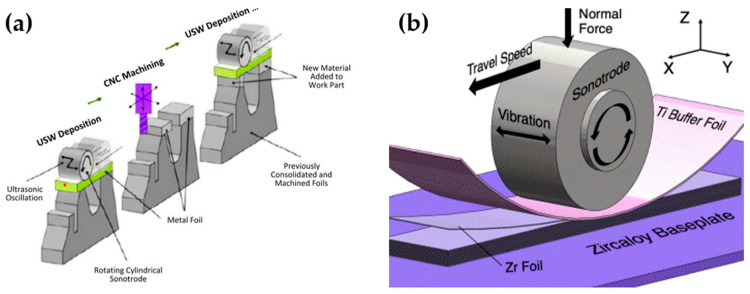
(**a**) Schematic of ultrasonic additive manufacturing; (**b**) ultrasonic additive manufacturing deposition tool [191,192].

UAM is a solid-state additive manufacturing process in which ultrasonic oscillation is utilized to fabricate 3D objects by layering thin metal foils. After layers of deposition, CNC machining is applied to create the shape of the object. Figure 36a illustrates the mechanism of UAM, and Figure 36b presents a UAM deposition tool setup. 

To better understand the dynamics of UAM, Hehr et al. [193] proposed a liner time-invariant model. The shear force and welder efficiency were successfully estimated, and process parameters were examined. The welder velocity was found to significantly affect the welder magnitude and efficiency. Levy et al. [194] explored the mechanism, post-processing, and properties of the UAM of steel. In this study, a laminated low-alloy carbon steel component was manufactured. Defects were reduced by applying SPS and HIP post-treatments, and the shear strength was significantly higher than that of as-printed objects. Gussev et al. [195] studied the influence of HIP on UAM samples and found that the HIP process improved z-direction strength and ductility; voids and defects were eliminated as well. 

**Figure 37:**
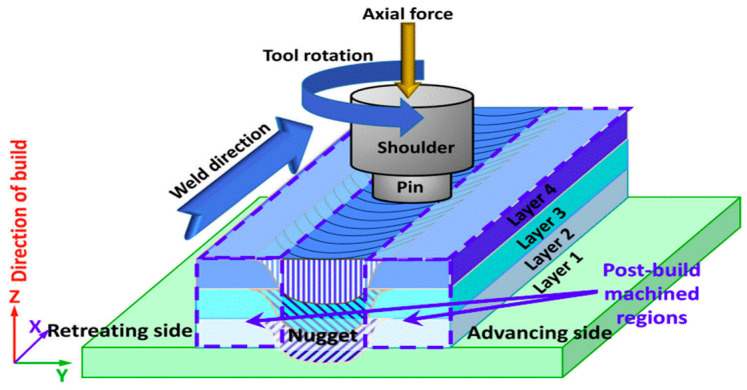
Schematic of friction stir additive manufacturing (FSAM) [196].

**Figure 38:**
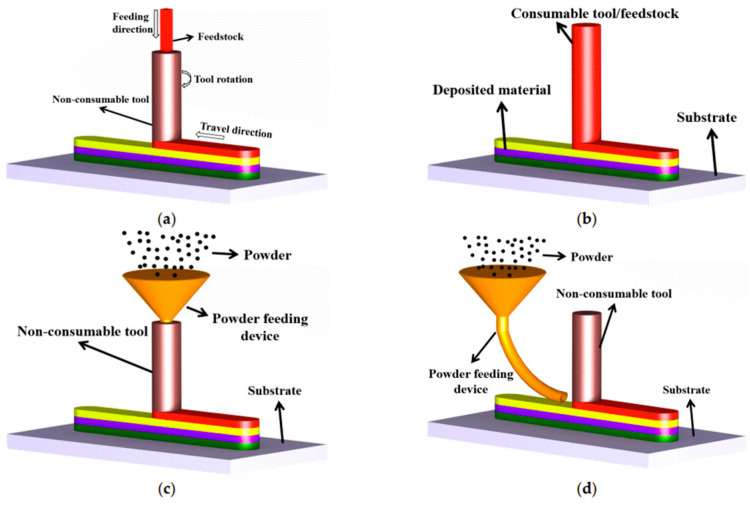
Schematic of additive friction stir deposition processes. (**a**) Non-consumable tool using rod as feedstock; (**b**) consumable tool; (**c**) non-consumable tool using powder as feedstock; (**d**) non-consumable tool using powder as feedstock with [199].

Figure 37 illustrates the schematic of the FSAM process. A non-consumable rotational tool is deployed on the overlapping metal sheets and travels along the joint trajectory. Additional machining is required to remove the unbonded side area. During the process, heat is generated due to friction between the tool and the metal sheets, leading to the metal sheets bonding. Similar mechanisms, such as AFSD, have been studied by various researchers, as shown in Figure 38. 

Using recycled metallic chips as the feeding material, Agrawal et al. [197] was able to create Ti64 components through AFSD. Hang et al. [59] explained the mechanism of AFSD. Instead of bonding existing metal foils, this novel approach applies continuous material feedstock filling and tool rotation processes, as shown in Figure 38a. Instead of vertical feedstock feeding, the filler material can also be fed from both sides, as studied by Haridas et al. [198]. 

Researchers have made efforts pertaining to the process control and applications of FSAM. Hassan et al. [200] summarized the processing parameters in FSAM in detail, including the machine-concerned, tool-concerned, material-concerned parameter, and other parameters. The microstructure and mechanical properties of FSAM builds were investigated as well. Khodabakhshi et al. [201] reviewed potential applications of FSAM, including power and rod feedstock-based FSAM processes and combinations of CSAM and FSAM. 

### 5.2. Materials, Mechanical Properties, and Defects of SL: The State of the Art

The summary of materials and print quality reported in the literature is presented in Table 5. 

**Figure 39:**
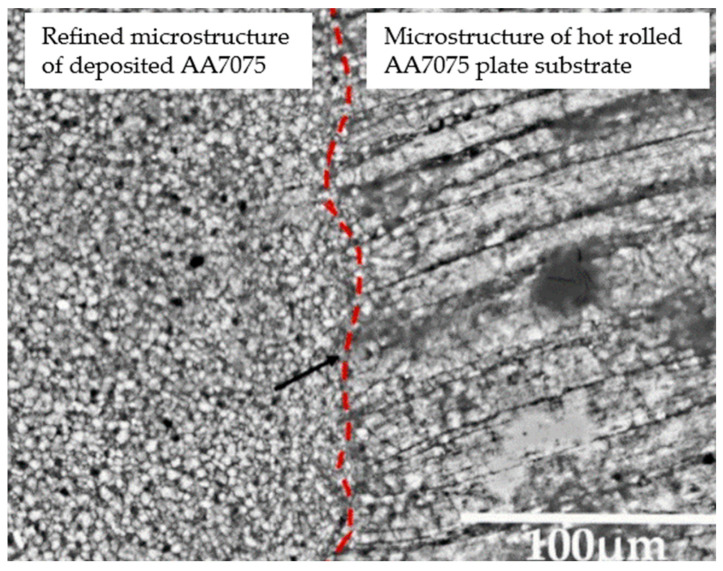
Microstructural comparison between deposited AA7075 alloy and a hot-rolled AA7075 plate. Red dash line indicates the hole wall edge [213].

Process monitoring and control are critical. Nadimpalli et al. [211] discovered an ultrasonic nondestructive evaluation method for monitoring and repairing defects in UAM, and a friction stir process was applied to repair common UAM defects. Qiao et al. [212] developed an in situ process monitoring device to read the real-time temperature, force, and torque during AFSD. The correlations between layer thickness and microstructure evolution/mechanical properties were studied based on the acquired data. To achieve enhanced mechanical properties, Zhou et al. [205] introduced an electropulsing process to UAM and found that the updated samples exhibited higher YS and UTS, with percentages of ~28 and ~26%, respectively. Xiao et al. [208] explored the process control of ASFD and found that the additive zone’s volume increased with a higher tool rotation speed; the tensile strength of the ASFD component was first enhanced as the tool’s transverse speed increased to 90 mm/min, while further increase led to tensile strength loss. He et al. [209] explored the evolution of microstructure and properties in ASFD and found that an overlapping interface may compromise the mechanical properties of the build; kissing bond defects were found to be mainly located at the overlapping positions. For microstructure refinement, Griffiths et al. [213] utilized FSAM in repairing volume damages such as through-holes in 7075 aluminum alloy. The filled AA7075 alloy exhibited an equiaxed and refined microstructure with an average grain size of 3.4 ± 0.7 μm. Figure 39 presents the refined microstructure of AA7075 alloy using FSAM. 

### 5.3. Wire-Based FSAM

**Figure 40:**
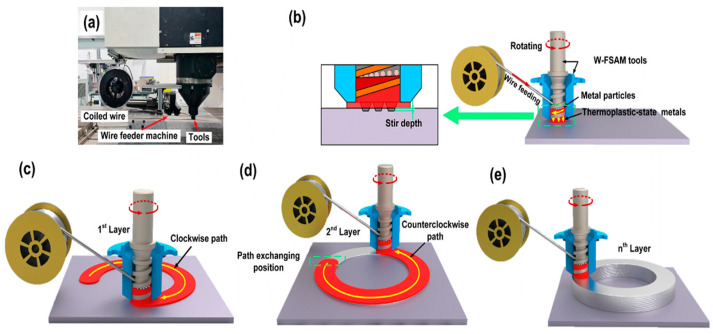
(**a**) Experimental setup for the wire-based FSAM; (**b**) dwelling section process; (**c**) 1st layer fabrication with a clockwise path; (**d**) 2nd layer fabrication with a counterclockwise path; (**e**) repeated process for the multilayer process [52].

Manufacturing Method: To address the current limitations of SL, Chen et al. [52] proposed an updated mechanism named wire-based FSAM. The wire-based FSAM setup features a fixed storage compartment with a port for the feeding wire, a transport mechanism with a screw design, and three mixing probes, as shown in Figure 40. The wire material was fragmented into metal particles by the screw-shaped transport mechanism and consistently moved downward. In the dwell section, these metal particles were persistently compressed and transformed into a thermoplastic state through the same screw-shaped transport system, keeping them in a ready-to-deposit state within the storage chamber [52].

Materials and Product Quality: Al-Si alloy was used as the printing material. This alloy is well known for its excellent thermal conductivity and low density, making it a suitable material for engine components [214]. Recently, this alloy was also used to manufacture electrode substrates for next-generation solar cells [215].

**Figure 41:**
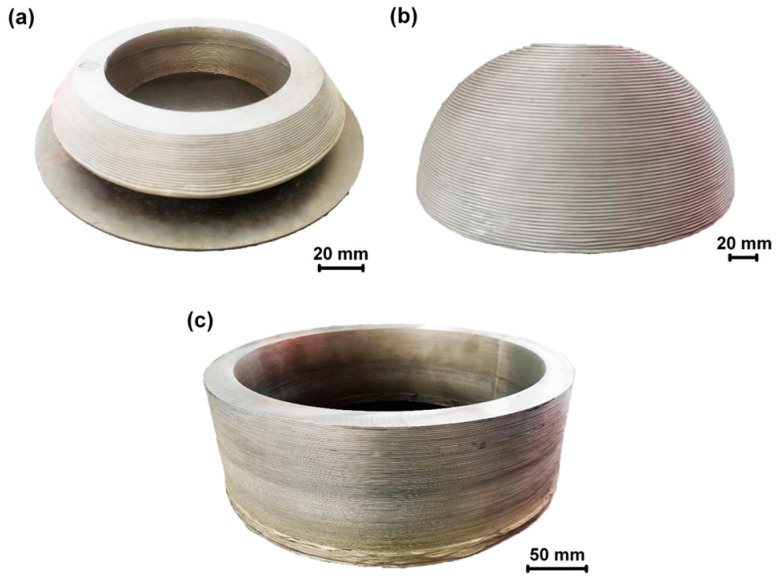
Three-dimensional (3D) objects fabricated by wire-based FSAM: (**a**) cone-shaped cylinder; (**b**) hemispheroid; (**c**) cylinder [52].

Figure 41 shows multilayer 3D structures obtained using wire-based FSAM. The thermo-plasticized materials were consistently pushed downward through a screw-like transport mechanism. At the same time, a fixed shoulder at the bottom of the storage chamber intensified the forging impact on these materials. Additionally, the agitation caused by the probes increased the dynamic fluidity of the thermo-plasticized materials, enhancing the metallurgical connection between the layers [52].

**Figure 42:**
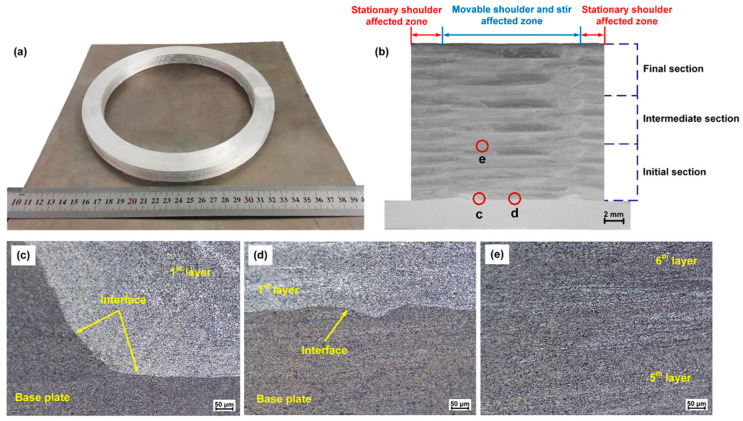
(**a**) Cylinder; (**b**) cross-sectional view of the cylinder; (**c**–**e**) interface bonding in points c, d, and e [52].

Adequate adhesion is beneficial for dimensional resolution during the printing process. As shown in Figure 42, the interfaces where bonding occurred showed a notably flat and straight appearance, suggesting that the thermo-plasticized materials from the deposited layer effectively blended with the base plate [52].

Summary: This study presents a wire-based FSAM process. The printing quality and resolution results meet the proposed requirements. Detailed analysis also illustrated the solid bonding between each layer. In addition, defects were not observed in the cross-sections of the printed samples. Nevertheless, the printed samples exhibited a high-dimensional geometry due to the print tool’s size, and they could be improved in further research.

## 6. Conclusions

This paper comprehensively reviews the diverse methods and recent advancements in MAM, mainly focusing on non-powder-bed-based MAM techniques. While powder-bed-based methods, such as PBF and BJ, continue to dominate the market, non-powder-bed-based approaches like DED, ME, and SL present promising alternatives. These techniques offer unique benefits, including larger build sizes and reduced energy consumption, but also face challenges like high residual stress and print defects. Moreover, print resolution and quality are two emerging challenges in non-powder-bed-based MAM. Innovations such as liquid-metal-assisted DED and hot-forge WAAM significantly enhance temperature and stress control, mechanical properties, and process efficiency, reducing the need for extensive post-processing and improving print quality. Ultrasonic-assisted DMW addresses the issue of inconsistent flow in the traditional DMW process; however, qualified 2D surfaces or 3D object samples were not presented or found. This might be due to liquid metal’s characteristics, including high surface tension and low viscosity. Thixotropic ME focuses on controlling the print material’s viscosity within the semisolid window, thereby improving the print resolution; meanwhile, thixotropy is beneficial in maintaining the printed material’s geometry compared to DMW, and several 2D surface print results in Lima’s study successfully prove this concept. Nonetheless, a consistent and uniform inter-filament adhesion remains challenging, and the inter-layer adhesion in thixotropic ME remains questionable. UAM has excellent advantages in multi-material fabrication. Due to the continuous pressing, shearing, and deposition processes, components printed using FSAM exhibit fewer defects and better mechanical properties. 

In summary, this paper comprehensively reviews recent developments and ongoing research in non-powder-bed-based MAM, highlighting its transformative potential in the manufacturing sector for more efficient, cost-effective, and flexible production of industrial components. However, this review is limited by the scarcity of related publications on ME and SL. There remains a research gap in exploring the mechanical performance of DMW, and there is a lack of cost analysis for SL. Future investigations should primarily focus on the ME and SL fields, with continued exploration and study of related mechanisms, material choices, cost analysis, and print quality.

## Figures and Tables

**Table 1:** Characteristics of direct energy deposition (DED), material extrusion (ME), and sheet lamination (SL).

DED		ME		SL	
Unlimited build size	[32]	Flexible build size	[33]	Large build size	[31]
High build rate with challenging process control, high residual stress, poor surface roughness, and low print resolution	[30,33,34,35,36,37,38]	Lower build rate with limited print resolution (depending on nozzle radius), support material needed, anisotropy of properties, and reduced residual stress	[33,34,39]	High volumetric build rate with high mechanical properties; ability to avoid solidification defects	[40,41]
Various material choices and multi-material print	[42,43,44]	Various material choices and multi-material print	[45,46,47,48]	Limited material choices and multi-material print	[32,33,49]
Energy consumption can be high, depending on process parameters	[50]	Less energy consumption	[51]	Less energy consumption; high machining cost with material waste	[32,52]
Suitable for repairing high-value components; need for high-value laser or electron beam; safety concerns of metal powder	[53,54,55]	Suitable for rapid prototyping; affordable cost; simpler and safer mechanism	[45,56,57,58]	Suitable for large geometry, affordable cost, and safe mechanism	[31,59]
Post-processing required	[33]	Additional debinding and sintering process; shrinkage due to post-processing	[27]	Additional machining processes	[41,59]

**Table 2:** Manufacturing cost per ${mm}^{3}$ for DED and ME [51,66,67].

Cost (USD)	DED		ME
	LDED	WAAM	MFFF
Machine	204	23.5	89.9
Material	66.7	4	31
Consumable	12.8	0.44	24.6
Post-processing	-	9.33	-
Labor	45.5	29.3	100
Energy	4.6	-	-
Equipment	4.9	-	-
Debinding	-	-	11.6
Sintering	-	-	70.4
$\mathrm{Sample}\;\mathrm{volume}\;({mm}^{3}$)	39,366	33,781	380,952
Total production cost	339	66.6	327.5
$\mathrm{Production}\;\cos t\;\mathrm{per}\;{mm}^{3}$	8.6 × 10^−3^	1.97 × 10^−3^	8.6 × 10^−4^

**Table 3:** Materials and print quality for DED reported in the literature.

Material/Alloy	DED Technologies	Mechanical Properties			Defects	Ref.
		Elongation (%)	YS (MPa)	UTS (MPa)		
Ti6Al4V	LDED	8	932	990	No defects observed	[83]
Ti65		9.1	~920	1058	-	[84]
Al-Cu		9.48 ± 0.12	182.8 ± 5.5	237.4 ± 7.2	Minor porosity and cracks	[63]
Al-Si		7.4 ± 0.5	187 ± 1.5	314 ± 2.4	Lack of fusion	[85]
Al7075		9.2 ± 1.1	271.8 ± 3.9	401.6 ± 1.9	Cracks and porosity	[86]
316L stainless steel		45.5	396	682	Porosity	[87]
IN625		23.14	675.83	1020.93	Lack of fusion	[88]
Cu-Fe		4.15	328	393	No defects observed	[89]
W50(Cobalt)		1.9 ± 0.2	-	646.1 ± 30.8	Porosity and cracks	[90]
Ti-6Al-4V	EB-DED	4.5	846	953	Lack of fusion, bonding defect	[91]
Ti60		7.1	863	932	-	[92]
In718		21 ± 4	655 ± 66	984 ± 75	Cracks	[93]
Al-Mg		33 ± 3.5	121 ± 7	253 ± 5	Porosity, shrinkage	[94]
Low-carbon Ni-Ti		6.1	450	534	No defects observed	[95]
High-carbon Ni-Ti		12.8	316	494	No defects observed	[95]
Ti-6Al-4V	WAAM	8.5	839	917	Collapse and cracks	[96]
TC17		5.9	1007	1049	Porosity and cracks	[97]
Al-Cu		8.12	101.2	232.99	Porosity	[98]
AZ31		26.2	102.6	239.8	Porosity	[99]
Mg–Li		12.4	-	201.4	Microporosity	[100]
IN625		~55	~430	~750	-	[101]
NiTi		3.7 ± 0.7	-	232 ± 11	No defects observed	[102]
Ti-6Al-4V	CSAM	~7	~1050	1110	Porosity	[103,104]
Copper		3 ± 1.5	-	191 ± 9	Voids, fracture, and porosity	[105]
Al6061		5.1–6.1	-	238–241	Porosity	[106]
Zn		18.4	-	92.2	No defects observed	[107]
316L stainless steel		-	-	~420	Porosity	[108]

**Table 4:** Materials and print quality for ME reported in the literature.

Material	ME Technologies	Mechanical Properties			Defects	Ref.
		Elongation (%)	YS (MPa)	UTS (MPa)		
Ti-6Al-4V	MFFF	17 ± 3	745 ± 10	875 ± 15	Small porosity	[39]
IN718		6.6 ± 0.5	-	1247 ± 140	Porosity	[157]
Al7075		1–2.5	-	120–140	Porosity	[158]
17-4PH SS		3.3	781	1018	Voids	[159]
316L SS		29.5 ± 3.8	194 ± 19	441 ± 27	Porosity	[160]
Copper		50.49	31	182	Voids	[159]
Ni-Ti		9.2	476	583	Porosity	[161]
Bi75-Sn25	DMW	-	-	-	No obvious defects	[152]
Sn63-Pb37		~25	-	92.2	Porosity	[151]
In61-B26-Sn9-Ga4		-	-	-	-	[162]
Sn63Pb37		-	-	-	Porosity	[163]

**Table 5:** Material and print quality for SL reported in the literature.

Material	SL Technology	Mechanical Property			Defects	Ref
		Elongation (%)	YS (MPa)	UTS (MPa)		
Zr	UAM	38.2 ± 3.5	366 ± 28	458 ± 24	Interfacial porosity	[192]
Al6061		-	-	-	-	[202]
		6	221	224	Microvoids	[203]
Ti/Al laminated		26.4 ± 0.4	257 ± 3	279 ± 1	Defect-free	[204]
Cu/Al laminated		4.3	261	285	Interfacial segregation	[205]
Cu/SS laminated		1.61%	-	165	Microcracks	[206]
Al7075	FSAM	8.2	477	541	-	[207]
Al6061-T6		32.8 ± 1.6	122 ± 1	214 ± 3	Defect-free	[198]
Al2024		19	-	475	Microvoids	[208]
Ti-6Al-4V		7 ± 1	1050 ± 25	1140 ± 20	Interfacial segregation	[197]
Al-Zn-Mg		8.4–22.3	-	352.7	Kissing bond defects	[209]
AISI4340 steel/AISI316L		11.2	1224	1912	-	[210]

## Abbreviations

MAM	Metal additive manufacturing
PBF	Powder bed fusion
BJ	Binder jetting
DED	Direct energy deposition
ME	Material extrusion
SL	Sheet lamination
LDED	Laser direct energy deposition
EB-DED	Electron beam direct energy deposition
WAAM	Wire-arc additive manufacturing
CSAM	Cold spray additive manufacturing
SR	Surface roughness
Ra	Roughness average
MIG	Metal inert gas welding
PAW	Plasma arc welding
TIG	Tungsten inert gas welding
YS	Yield stress
UTS	Ultimate tensile strength
MFFF	Metal fused-filament fabrication
EM	Extrusion multiplier
FFF	Fused-filament fabrication
DMW	Direct metal writing
FSAM	Friction stir additive manufacturing
UAM	Ultrasonic additive manufacturing
HIP	Hot isostatic pressing
PLA	Polylactic acid
IH	Induction heating
CNC	Computer numerical control
AFSD	Additive friction stir deposition
